# Evaluation of Anti-Inflammatory and Atheroprotective Properties of Wheat Gluten Protein Hydrolysates in Primary Human Monocytes

**DOI:** 10.3390/foods9070854

**Published:** 2020-06-30

**Authors:** Sergio Montserrat-de la Paz, Noelia M. Rodriguez-Martin, Alvaro Villanueva, Justo Pedroche, Ivan Cruz-Chamorro, Francisco Millan, Maria C. Millan-Linares

**Affiliations:** 1 Department of Medical Biochemistry, Molecular Biology and Immunology, School of Medicine, Universidad de Sevilla, Av. Dr. Fedriani 3, 41071 Seville, Spain; delapaz@us.es (S.M.-d.l.P.); icruz-ibis@us.es (I.C.-C.); 2 Department of Food & Health, Instituto de la Grasa, CSIC. Ctra. de Utrera Km. 1, 41013 Seville, Spain; noe91rm@gmail.com (N.M.R.-M.); alvarovillanueva@ig.csic.es (A.V.); jjavier@cica.es (J.P.); frmillan@cica.es (F.W.); 3 Instituto de Biomedicina de Sevilla, IBiS (Universidad de Sevilla, HUVR, Junta de Andalucía, CSIC), Av. Manuel Siurot s/n, 41013 Seville, Spain; 4 Cell Biology Unit, Instituto de la Grasa, CSIC. Ctra. de Utrera Km. 1, 41013 Seville, Spain

**Keywords:** wheat gluten, protein hydrolysates, atherosclerosis, monocytes, celiac disease

## Abstract

Bioactive protein hydrolysates have been identified in several sources as possible agents in the prevention and treatment of many diseases. A wheat gluten (WG) concentrate was hydrolyzed by Alcalase under specific conditions. The resulting hydrolysates were evaluated by in vitro cell-free experiments leading to the identification of one bioactive WG protein hydrolysate (WGPH), which was used at 50 and 100 μg/mL on primary human monocytes. Reactive oxygen species (ROS) and nitrite levels and RT-qPCR and ELISA techniques were used to analyze the functional activity of WGPH. Our results showed that WGPH hydrolyzed in 45 min (WGPH45A) down-regulated gene expression of Interleukin (*IL*)-*1β*, *IL*-*6*, *IL*-*17*, and Interferon gamma (*IFNγ*) and reduced cytokine release in lipopolysaccharide (LPS)-stimulated monocytes. In addition, WGPH45A down-regulated gene-related to atherosclerotic onset. Our results suggest that WGPH45A has a potent anti-inflammatory and atheroprotective properties, reducing the expression of gene-related inflammation and atherosclerosis that could be instrumental in maintaining cardiovascular homeostasis.

## 1. Introduction

Proteins are essential nutrients for the smooth operation organisms. The protein requirement in the diet is needed for several cell functions such as in the building and repairing of tissues, or such as for signaling molecules among others. Plant proteins are an interesting source to find new lead compounds of good-quality that are cheaper, and with a lesser environmental impact than animal proteins [1]. The substitution of animal proteins with plant proteins in the food industry or in our own diet is another important issue for health [2]. Within plant proteins, the study of their enzymatic hydrolysates is of special interest as, they have many bioactive functions, some of which have been shown to exhibit anti-inflammatory, antioxidant, or other bioactive activities [3,4,5,6]. 

Wheat is consumed worldwide and is a cultivated cereal and it is the basic food in the human diet through bread consumption. Wheat gluten (WG) is an economically important co-product in the recovery of wheat starch in wet milling of wheat flour [7]. Currently, wheat protein is used in animal and human nutrition. Approximately 80% of the production of wheat is for human consumption [8]. The main food application for gluten is bread, but it is also used to enrich other foods such as breakfast cereals, pasta, flour-based foods, and vegetable-based meat alternatives that are on the rise [8]. Gluten is a complex mixture of hundreds of related but distinct proteins, mainly gliadin and glutenin, which are highly resistant to hydrolysis and are mediated by proteases of the human gastrointestinal tract. Being able to generate oligopeptides, they remain in the small intestine for longer periods of time and can produce inflammation and consequently celiac disease and allergy in genetically predisposed people [9,10]. Nowadays, the increasing incidence of celiac disease has shifted the focus on to the development of WG-derivative healthy food ingredients [11]. WG enzymatic hydrolysis, which is essentially appropriate for expanding applications in food products, could produce functional WG protein hydrolysates (WGPHs). WG is composed of a high amount of glutamine and nonpolar amino acids (proline and glycine), which render the protein insoluble in aqueous media [12]. The low water solubility of gluten at a neutral pH limits its application as a functional component in the food industry, even though its cost is low compared to that of other protein isolates, such as those from peas, soy, or animal sources [13]. Several chemical and enzymatic modification strategies for improving the water solubility of gluten have been explored. Enzymatic hydrolysis tweaks the molecular mass and the openness of hydrophobic regions to the surrounding aqueous phase.

Atherosclerosis is a common multifactorial disease with an important nutritional role. It is responsible for a high fraction of cardiovascular diseases [14]. A feature of atherosclerosis is chronic inflammation and increased activation of monocytes in the systemic blood circulation. Monocytes are cells with a myeloid origin, which are actively engaged in systemic and chronic inflammation and have distinct contributions to the formation, progression, and destabilization of the atherosclerotic plaque [15]. The metabolism of monocytes can be reprogrammed in the atherogenic microenvironments, which consist of hypoxia, molecular damage, and modified lipoproteins [16]. It is widely known that monocytes have an important role in the early stages of atherosclerosis. Monocytes are recruited to the endothelial layer; monocytes squeeze into the wall and are differentiated into macrophages mediated by adhesion molecules and cytokines [17]. Human monocytes are classified as different phenotypes depending on the surface membrane antigen expression, which is especially important for the disease progression. The M1 phenotype highly expresses the CD14 antigen in the cell membrane and relates to the pro-inflammatory stage with inflammatory marker production. Alternatively, the M2 phenotype highly expresses the CD16 antigen in its membrane and relates to anti-inflammatory marker production and inflammation resolution. This plastic character may define the monocyte cells phenotype, which is predicted on the chemical environment. For instance, the atherogenic microenvironment highly expresses the M1 phenotype, which is related to unstable plaque formation, while an anti-atherogenic microenvironment expresses the M2 phenotype and is related to stable plaques (17). An endogenous material addition such as lipopolysaccharide (LPS) thrives in a negative environment of monocyte and triggers the activation of the M1 phenotype. Despite this, the negative environment can be reversed with treatment of bioactive natural food chemicals such as small peptides, which triggers the activation of the M2 phenotype. Activated monocytes express high levels of chemokine receptors (CCRs) infiltrating into the sub-endothelial space upon binding to the adhesion molecules on the endothelium surface [18]. In addition, activated monocytes also produce high amounts of reactive oxygen species (ROS) and pro-inflammatory cytokines, including tumor necrosis factor (TNF)-α, interleukin (IL)-1β, and IL-6 in response to LPS in vitro [19]. They also have a greater capacity for trans-endothelial migration, the ingestion of oxidized low-density lipoprotein (ox-LDL) via CD36 within the intima of the atherosclerotic lesion and become “foam cells” [20]. Taken together, the purpose of this work was to study the potential anti-inflammatory and atheroprotective activity of new WGPHs in primary human monocytes. 

## 2. Materials and Methodology 

### 2.1. Hydrolysis of Wheat Gluten Protein Concentrate 

Hydrolysis of WG protein concentrate (WGPC, chemical, and amino acidic characterizations are shown in Tables S1 and S2, respectively) was carried out in a bioreactor in uninterrupted stirring at a restricted pH and temperature. WGPC was suspended in distilled water (10% w/v) and pH and temperature were adjusted to 8 and 50 °C, respectively. Alcalase 2.4 L (2.4 AU/g was kindly donated by Novozymes, Bagsvaerd, Denmark) was added at enzyme/substrate = 0.3 AU/g protein. Samples were taken at 0, 15, 30, 45, and 60 min. After that, the resulting hydrolysates were boiled at 85 °C for 15 min in order to stop enzymatic reaction. The supernatants obtained after centrifuging at 19107.7 g for 15 min constituted the WGPHs. The resulting hydrolysates were composed of a mixture of amino acids and small peptides. The WGPHs were designated with the suffix: 0A, 15A, 30A, 45A, and 60A, which indicates in min the hydrolysis time.

### 2.2. Hydrolysis Degree Determination

The hydrolysis degree (HD), defined as the percentage of peptide bonds cleaved, was evaluated by the TNBS (Sigma-Aldrich, St. Louis, MO, USA) method according to Adler-Nissen (1979) [21]. The amino group total number was examined in a sample that had been 100% hydrolyzed at 110 °C for 24 h in 6 N HCl.

### 2.3. Inhibitory Activity of Angiotensin-Converting Enzymes

Angiotensin-converting enzyme (ACE) activity was evaluated in harmony with the method described by Sentandreu and Toldrá [22]. Briefly, 40 µL of WGPHs (at 1 µg/µL) were added to 40 µL of ACE (at 0.04 U/mL) in a 96-well multiplate. The addition of 160 µL of Abz-Gly-p-nitro-Phe-Pro-OH (Bachem AG, Bubendorf, Switzerland) started the reaction at 0.45 mM in 150 mM Tris-base buffer (pH 8.3) with 1.125 M NaCl. After 30 min of incubation at 37 °C, fluorescence was assessed using 340 and 535 nm as excitation and emission wavelengths, respectively.

### 2.4. Inhibitory Activity of Thrombin

Thrombin activity was evaluated according to Ialenti et al. [23] Briefly, 50 µL of WGPHs at a concentration of 20 µg protein/µL, was previously incubated at room temperature for 1 h with 5 µL of enzymes (at 0.25 U/µL). Then the mixture was resuspended in 1.45 mL of 50 mM Tris and 150 mM NaCl (pH 8.3). The addition of 0.5 mL of 0.3 mM Chromozym started the reaction. The increase of absorbance at 405 nm was obtained after 5 min. A mixture composed by 30 µL of AT III (4.04 µg/mL) and 15 µL of heparin (3.74 g/mL) was used as a positive control.

### 2.5. WGPH45A Characterization and Amino Acid Profile

In the selected hydrolyzed WGPH45A, after 45 min of hydrolysis with Alcalase of WGPI, the protein concentrations were assessed by elemental microanalysis as % nitrogen content × 6.25 using a Leco CHNS932 analyzer (St. Joseph, MI, USA). Total dietary fiber was obtained by the gravimetric method [24]. Ash content was measured according to the direct ignition method (550 °C for 36 h) and oil content was measured using the AOAC method 945.16 [25]. Polyphenols were determined using standard curves of chlorogenic acid, while soluble sugars were obtained using standard curves of glucose [26,27]. Amino acid quantification was determined according to the Alainz et al. method by High Performance Liquid Chromatography (HPLC) [28]. To finish, tryptophan quantification was measured according to the Yust et al. method [29].

### 2.6. Analysis of Molecular Profile by Fast Protein Liquid Chromatography (FPLC)

FPLC Akta purifier 10 (GE Healthcare Bio-sciences AB, Uppsala, Switzerland) was used in order to determined the molecular profile of each sample using the Superose 12 HR 10/300 GL colum, which is a prepacked column for high-performance size exclusion chromatography. Hence, the fractionation range broad for molecules with molecular weights between 1 and 300 kDa, was used. The column was previusly calibrated with different well-known and defined proteins. From the logarithms of the molecular weights of these proteins and their elution volumes, the calibration line was made. The elution was carried out using a flow of 1 mL/min with 50 mL of sodium phosphate buffer (0.05 M), sodium chloride (0.5 M), and sodium azide (0.02% w/v) at pH 7.5. The injected volume of each sample was 500 μL and its concentrations were of 1 mg/mL of protein. The elution of the proteins was reported at 280 nm of absorbance.

### 2.7. Isolation of Primary Human Monocytes

This study was managed according to the Good Clinical Practice Guidelines and in line with the principles delimited in the Helsinki Declaration of the World Medical Association. Blood was obtained from healthy donors in Centro Regional de Transfusion Sanguinea de Sevilla-Huelva y Banco de Tejidos. Firstly, the peripheral blood mononuclear cells (PBMCs) were isolated by centrifugation over a Ficoll-Histopaque (Sigma-Aldrich, Madrid, Spain) gradient from buffy coat. CD14 microbeads and LS columns on a midiMACS system (Miltenyi Biotec, Madrid, Spain) were used in order to isolate monocytes from PBMCs according to the specified protocol of the manufacturer. The purity for CD14 monocyte isolations was routinely >95% by flow cytometry (FACScanto II flow cytometer and FACSDiva software, versión 5.0.1., Becton Dickinson, Erembodegem, Belgium) [30]. After that, monocytes were cultivated in a supplemented (L-glutamine, 1% penicilin/streptomicin, 10% foetal bovine serum) Roswell Park Memorial Institute (RPMI) 1640 medium (Sigma-Aldrich, St. Louis, MO, USA). Finally, the in vitro treatments were performed over purified monocytes in a density of 5 × 10^5^ in a 12-well plate. Monocytes were previously stimulated with or without 0.1 µL/mL of LPS, and were exposed to WGPH45A for 24 h at 50 and 100 µg/mL.

### 2.8. Cell Viability Assay (MTT)

Monocytes were seeded in 96-well plates at a density of 1 × 10^4^ cells/well and incubated with WGPH45A at different concentrations up to 200 µg/mL for 24 h. Next, the MTT solution (Sigma-Aldrich, St. Louis, MO, USA) was added to each well until a purple precipitate was visible. MTT-formazan crystals were finally solubilized with DMSO (Sigma-Aldrich, St. Louis, MO, USA). The outcomes were obtained in a microplate reader at 570 nm corrected to 650 nm [31]. Cell survival was calculated as the percentage of absorbance contrasted with that obtained in control, non-treated cells.

### 2.9. Reactive Oxygen Species (ROS) Generation

The intracellular ROS was obtained using the CellROX Green Reagent (ThermoFisher Scientific, Madrid, Spain). After in vitro stimulation with LPS at 100 ng/mL, primary human monocytes were exposed to 50 μg/mL and 100 μg/mL of the WGPH45A for 24 h and then with CellROX Green Reagent (5 μM) for 30 min. Cells were washed with PBS and fixed with 3.7% formaldehyde, and the fluorescence signal was analysed in a Fluoroskan Microplate Fluorometer (ThermoFisher Scientific) equipped with a 485/555 excitation/emission filter set. The auto-fluorescence of cells was measured under the same conditions but without adding CellROX Green Reagent. Data shown refers to the % of intracellular ROS production and to the comparison with a positive control (100% ROS production) after cell treatment in the presence of LPS.

### 2.10. Nitic Oxide (NO) Generation

Primary human monocytes (10^5^ cells/well in 24 well plates), after being stimulated with LPS at 100 ng/mL, were incubated for 24 h in the existence of 50 μg/mL and 100 μg/mL of WGPH45A. The Griess reagent (Sigma-Aldrich) was used to calculate the production of nitrite, considered an NO generation indicator. We had previously transferred 100 μL of the culture supernatant to a 96 well plate, and a volume of 100 μL of Griess reagent was added. A BioTek plate reader measured absorbance at 540 nm wavelength, using a sodium nitrite standard curve to estimate concentration. 

### 2.11. RNA Isolation and qRT-PCR Analysis 

RNA was totally extracted by using Trisure Reagent (Bioline GmbH, Luckenwakde, Germany), following the manufacturer’s instructions. The measure of A_260_/A_280_ ratio in a NanoDrop ND-1000 Spectrophotometer (Thermo Scientific, Madrid, Spain) was used to determinate the RNA grade. Then, the reverse transcription (iScript, Bio-Rad, Madrid, Spain) was performed to obtain cDNA from RNA (1 µg). The resulting cDNA (10 ng) was used as a template for real-time PCR amplifications. The mRNA levels for specific genes were determined in a CFX96 system (Bio-Rad). For each PCR reaction, a cDNA template was added to Brilliant SYBR green QPCR Supermix (Bio-Rad, Hercules, CA, USA) containing the primer pairs for either genes or for housekeeping genes such as glyceraldehyde 3-phosphate dehydrogenase (GAPDH) (Table S3). All amplification reactions were performed at least thrice and average threshold cycle (Ct) numbers of these triplicates were used to obtain the relative mRNA expression of candidate genes. The magnitude of change of mRNA expression for candidate genes was calculated by using the mathematical method of 2^−(ΔΔCt)^. Briefly, all candidate genes were normalized to housekeeping genes (GAPDH) and expresed as the control’s percentage.

### 2.12. Cytokine Quantification

The levels of IL-1β, IL-6, IL-17, and IFNγ in culture supernatants were determined by enzyme-linked immunosorbent assay (ELISA), following the protocol of the manufacturer (Thermo Fisher Scientific, Waltham, MA, USA). The cytokine concentrations were expressed in pg per mL for IL-1β, IL-6, and IL-17 and in ng per mL for IFNγ, as calculated from the calibration curves from serial dilution of human recombinant standards in each assay. 

### 2.13. Statistical Analysis

All analysis was performed in triplicate. The outcomes obtained are mathematically represented as arithmetic means ± standard deviations (SD) and assessed with Graph Pad Prism Version 5.01 software (San Diego, CA, USA). The one-way analysis of variance (ANOVA) was used to evaluated the statistical significance of any difference in each parameter among the groups, also the Tukey multiple comparisons test was used as a post hoc test. *P* values considered statistically significant were less than 0.05.

## 3. Results and Discussion

**Figure 1:**
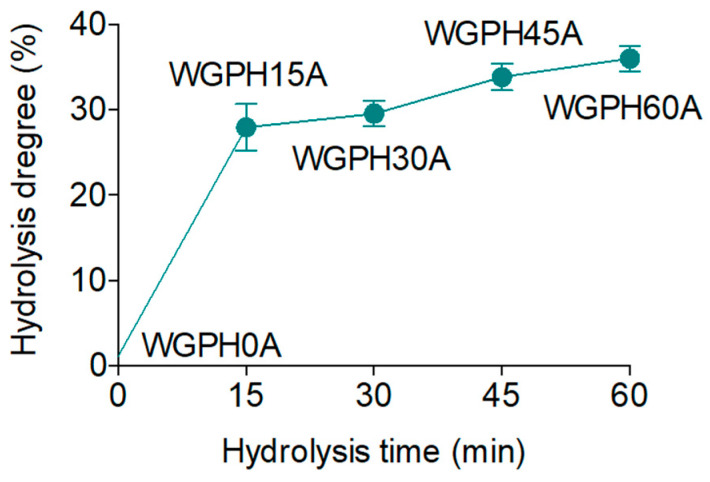
Time-course of Hydrolysis Degree (HD) of WGPC (WGPH0A) during hydrolysis with enzyme Alcalase at 15 (WGPH15A), 30 (WGPH30A), 45 (WGPH45A), and 60 (WGPH60A) min. All values are expressed as the percentage of peptide bonds cleaved, and are mean ± standard deviation of triplicates.

The hydrolysis length may be assessed by the HD, which also can indicate longer or shorter lengths of the peptide chain. Previous studies showed <30% values of HD in others protein hydrolysates by the use of a single enzyme [7]. The HD outcomes of WGPC treated with Alcalase after 0, 15, 30, 45, and 60 min is shown in Figure 1. Analysis of results showed that Alcalase performance increased up to 28% the HD in 15 min (WGHP15A), rising up to 36% after 60 min (WGHP60A) of hydrolysis. A high reaction rate has also been showed for the hydrolysis of other vegetable proteins using Alcalase [32,33]. 

**Figure 2:**
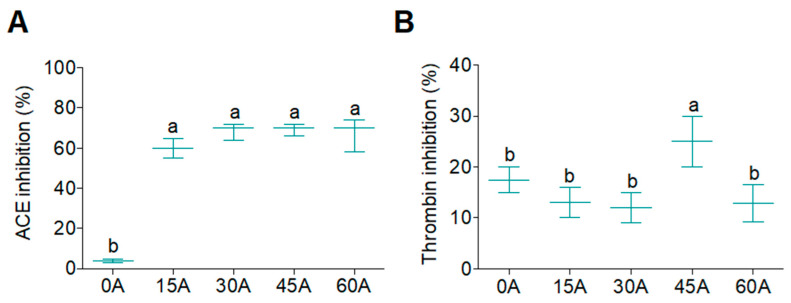
Inhibition of the angiotensin-converting enzyme (ACE) (**A**) and thrombin (**B**) activity by WGPHs performed using Alcalase in cell-free in vitro experiments. Data are showed as means ± SD (*n* = 3) and those marked with different letters are significantly different (*p* < 0.05).

The resulting hydrolysates were evaluated by in vitro cell-free experiments leading to the identification of one bioactive WGPH. ACE activity, which plays an important role in regulating blood pressure in the renin-angiotensin system, atherosclerosis, and inflammation [34], was determined in the presence of WGPHs. The ACE-inhibitory activity of WGPHs are reported in Figure 2A. Whereas WGPC (WGPH0A) did not show ACE inhibition, all WGPHs reduced ACE activity by more than 60%. These results are better than those obtained by Motoi and Kodama [35] who hydrolyzed WG with six different proteases, not including Alcalase, and found ACE inhibition rates between 17% and 52%. In fact, Alcalase has been previously reported to be the most effective protease to yield ACE-inhibitory peptides [36,37]. In addition, thrombin, which exerts the formation of blood clots, pro-inflammatory effects by stimulating the production of pro-inflammatory cytokines, and the onset of atherosclerosis [38] was determined in order to evaluate the thrombin-inhibitory activity of WGPHs (Figure 2B). WGPC (WGPH0A) inhibited 18.5% of thrombin activity. This result was only improved with Alcalase hydrolysis after 45 min (WGPH45A), which showed 25% thrombin-inhibitory activity. Although this value is not very high, similar results with Alcalase were reported in lupine protein hydrolysates [33], and in blue mussel proteins [39]. Taking together HD, ACE, and thrombin-inhibitory activity, we selected WGPH45A to analyze chemical and amino acidic composition and to evaluate biological activity in primary human monocytes.

The chemical composition of WGPH45A is reported in Table 1. The main difference it has with WGPC is the increase in ash content, which is due to the addition of NaOH to maintain a constant pH during the hydrolytic process, a usual result after the hydrolysis of several protein sources [40]. The amino acid content in WGPH45A is reported in Table 2. WGPH45A showed 38% glutamic acid/glutamine, 7% leucine, and 7% proline. WGPH45A complies with Food and Agriculture Organization/World Health Organization (FAO/WHO) recommendations for branched amino acids as leucine, isoleucine, and valine, and sulfur amino acids such as methionine and cysteine. In addition, histidine content exceeds what is recommended (> 1.9). Several studies have shown that a diet rich in histidine can help suppress the pro-inflammatory response. For instance, histidine-rich supplementary meals analyzed in vivo have shown a sepsis status improvement by the inhibition of systemic inflammation [41]. Besides this, histidine-dipeptides and small peptides rich in histidine may have the ability to reduce oxidative stress in chronic disease [42]. Additionally, plasma protein that is rich in histidine was negatively associated with oxidative stress and inflammation in a clinical trial with obese women [43] and was also associated with a general inflammatory status, which relates to the pathophysiological role of this amino acid [44]. 

**Figure 3:**
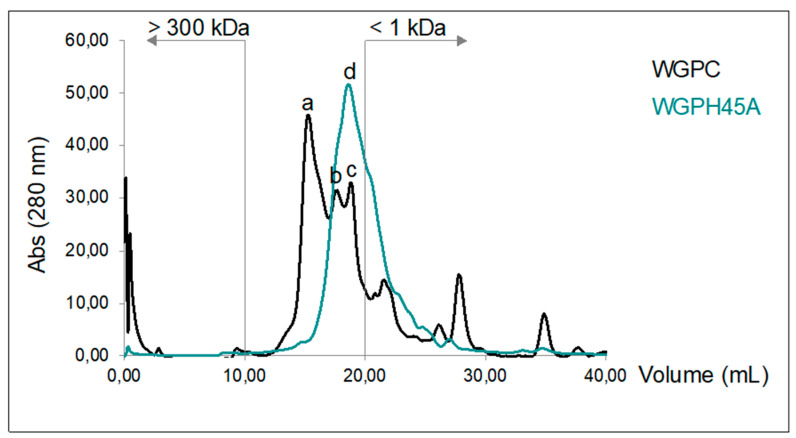
Molecular weight profiles by size-exclusion Fast Protein Liquid Chromatography (FPLC) of WGPC and WGPH45.

As depicted in Figure 3, the analysis of the protein molecular profile by HPLC determined that WGPC is constituted mainly by peptides of three sizes in the range of 1–300 kDa (30.08, 5.36, and 2.19 kDa). The WGPH45A protein molecular profile showed that all peptides are in a value next to 1.90 kDa. Results indicated that enzymatic hydrolysis by Alcalase clearly reduced the high molecular weight of WGPC, and that WGPH45A obtains low molecular weight peptides (<2 kDa). Reportedly, low molecular weight peptides (2–20 amino acids) are more biologically active compared to their parent polypeptide/proteins, which are larger [45].

**Figure 4:**
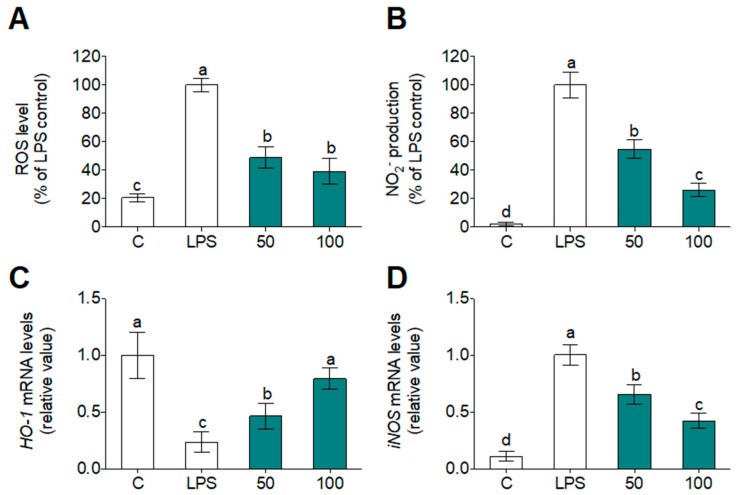
Intracellular ROS (**A**) and nitrite (**B**) production, expressed as a percentage of fluorescence/absorbance, and *HO-1* (**C**) and *iNOS* (**D**) mRNA levels after 24 h incubation with or without LPS (100 ng/mL) and WGPH45A at 50 and 100 µg/m. Values are presented as means ± SD (*n* = 3) and those marked with different letters are significantly different (*p* < 0.05).

The inflammatory stage has been associated with several chronic diseases related to cardiovascular and atherogenic risk and the activation of circulating blood cells, as primary human monocytes, is an accepted method to validate the in vitro inflammation assays [19]. Oxidative stress and ROS production are directly implicated at the beginning of inflammatory progression. Together, the oxidative stress and specific pro-inflammatory molecule release allows the triggering of events involved in the atherosclerosis origin, such as circulating lipoprotein oxidation from the bloodstream [20]. In previous studies, plant protein hydrolysates have exhibited anti-inflammatory activities in the THP-1 cell line [46] and primary human monocytes [30]. Herein, to our knowledge, we now report for the first time that a new WGPH has the ability to prevent the pro-inflammatory activation of primary human monocytes. Cells were incubated with WGPH45A at concentrations up to 200 µg/mL for 24 h. The cell viability evaluated by the MTT method was not affected by any WGPH45A concentration (data not shown). Quantification of both ROS (Figure 4A) and nitrite (Figure 4B) concentrations in primary human monocytes let us analyze the preventive role of WGPH45A on oxidative conditions. LPS remarkably increased both intracellular ROS and nitrite, compared to non-stimulated cells. The ROS and nitrite production induced in the presence of WGPH45A was significantly lower than LPS control. In addition, LPS down- and up-regulated Heme oxygenase (*HO)-1* (Figure 4C) and inducible Nitric oxide synthase (*iNOS)* (Figure 4D) mRNA expression, respectively. Interestingly, *HO-1* levels are lowly expressed in macrophages that are enriched in progressing plaques [47]. Recently, rice bran protein hydrolysates have been shown to restore *HO-1* expression [48]. We have no evidence of previous studies regarding the effect of WGPHs on *HO-1* and *iNOS* expression; however, gluten-related disorders are mediated by an up-regulation of *iNOS* [49]. WGPH45A could be an appropriate food ingredient in celiac patients.

**Figure 5:**
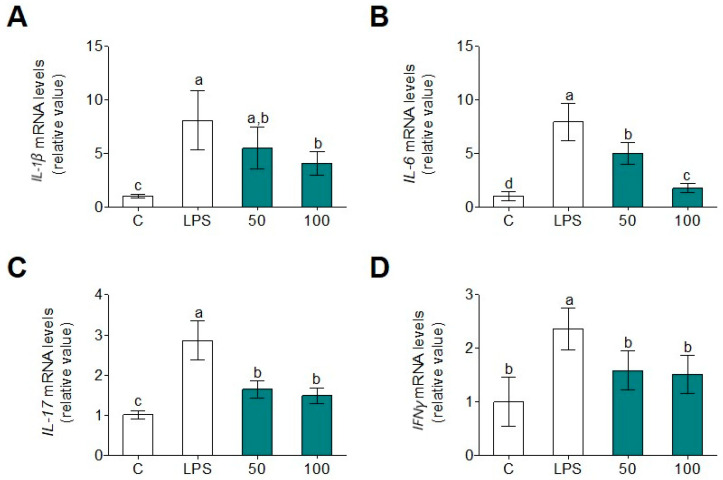
Gene expression of *IL-1β* (**A**), *IL-6* (**B**), *IL-17* (**C**), and *IFNγ* (**D**) in primary human monocytes after 24 h incubation with or without LPS (100 ng/mL) and WGPH45A at 50 and 100 µg/m. Values are presented as means ± SD (*n* = 3) and those marked with different letters are significantly different (*p* < 0.05).

**Figure 6:**
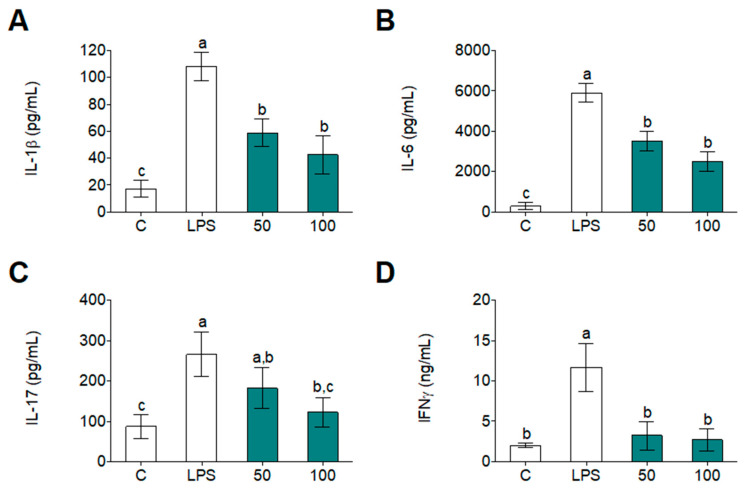
Cytokine secretion of IL-1β (**A**), IL-6 (**B**), IL-17 (**C**), and IFNγ (**D**) in primary human monocytes after 24 h incubation with or without LPS (100 ng/mL) and WGPH45A at 50 and 100 µg/mL. Data are expressed as means ± SD (*n* = 3) and those marked with different letters are significantly different (*p* < 0.05).

In gaining a deeper insight into the role of WGHPs on human monocyte inflammation, we observed that LPS induced *IL-1β*, *IL-6*, *IL-17*, and *IFNγ* gene expression (Figure 5) and release of these pro-inflammatory cytokines (Figure 6) were diminished by WGPH45A in LPS-treated primary human monocytes. The cytokine environment of these monocytes could indicates that M1 phenotypes related to pro-inflammatory progression had been lowered, while M2 phenotype, which is associated with resolution and tissue repairs, had been enhanced. In the literature, it possible to find a large number of examples where plant-derived protein and peptides have been used as anti-inflammatory or antioxidant compounds. In 2017, Kan et al. demonstrated that a combination of wheat peptides and fucoidan attenuates oxidation and inflammation of ethanol-induced gastric mucosal damage in rats [50]. Other protein hydrolysates isolated from nutritional seeds have also showed anti-inflammatory properties. For instance, the case of hemp seed (*Cannabis sativa* L.) protein hydrolysates, which inhibited LPS-induced inflammation in the BV2 cell line [6], such as in the case of amaranth (*Amaranth hypochondriacus*) hydrolysates, which also inhibited LPS-induced inflammation in human and mouse myeloid cells by preventing activation of NF-κB signaling [51]. Our study can improve the efficacy of existing WGPHs that may delay or prevent immune- and inflammatory-related diseases.

**Figure 7:**
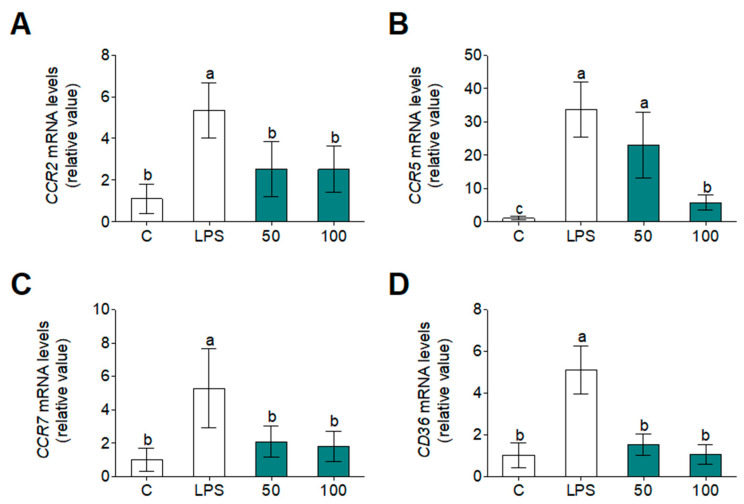
Gene expression of *CCR2* (**A**), *CCR5* (**B**), *CCR7* (**C**), and *CD36* (**D**) in primary human monocytes after 24 h incubation with or without LPS (100 ng/mL) and WGPH45A at 50 and 100 µg/mL. Data are expressed as means ± SD (*n* = 3) and those marked with different letters are significantly different (*p* < 0.05).

Finally, the scientific literature has demonstrated that the inflammatory component of atherosclerosis is represented by various cells and mediators in the immune system [52]. The progression of the plaque formation was thrived by several inflammatory mediators, which change over the cell environment in the release of the above-mentioned cytokines (mainly IL-1β and IL-6) and induce CCR production that has been directly involved in plaque development. The transfer of monocytes into a plaque requires three sequential steps: the capture, rolling, and transmigration of monocytes. *CCL5* have an important role in the capture and rolling steps, which are attached to the luminal surface of the endothelium mediated by adhesion molecules (glycosaminoglycans, P-selectin [47]). *CCR2* are involved in the transmigration activity between vascular endothelial cells and intima layers. Additionally, *CCR7* promote migratory activity in LPS stimulated cells [53]. Apart from CCRs chemokynes, M1 phenotype macrophages have the ability to recognize and process modified LDLs by scavenger receptors such as *CD36* which bind to ox-LDL and differentiate to foam cells [47]. Hence, for the first time, a WGPH has showed atheroprotective properties in primary human monocytes. To observe atheroprotective properties of WGPH45A after LPS-stimulated monocytes, we extracted mRNA and performed RT-qPCR of atherosclerosis-related genes (Figure 7). LPS increased mRNA levels of CCRs (*CCR2, CCR5,* and *CCR7*) and *CD36* in primary human monocytes. Interestingly, the WGPH45A significantly suppressed the transcriptional activity of the CCR family and *CD36* genes. Chemokines have been implicated in promoting migration of monocytes into the arterial intima. Monocyte chemoattractant proteins attract monocytes bearing the chemokine CCRs. The uptake of ox-LDL is mediated by scavenger receptors, including *CD36*, promoting foam cell formation and atherosclerosis onset [54]. To our knowledge, we can now report for the first time that a new WGPH has the ability to prevent the activation of atherosclerosis-related genes in primary human monocytes.

Derived food peptides that have shown bioactive activity by several in vitro assays can be used to control different diseases and additionally they are safe and cost-effective bioactive compounds. However, the fact is that they can resolve not only from the transport into the intestinal epithelial membrane, but also from enzymatic degradation of intestinal proteases and its administration dose as several WGPH-related studies have indicated [55,56]. For instance, the literature evidence has shown resistant peptides that are available as egg-derived peptides [47]. The peptide stability and its resistance to protease degradation in the intestinal lumen has been substantially correlated with the non-polar proline residue content (WGPH45A, 7.12 ± 0.63 in proline) [47]. In addition, various peptides can be transported across the intestinal epithelial membrane. This is the case was derived-milk peptides or corn and wheat gluten hydrolysates, which have been identified in the human bloodstain after ingestion [57,58]. Common peptide transport routes are at circulating bloodstain carrier-mediated permeation, transcytosis, paracellular transport or passive diffusion. Critically, the paracellular route may be the best option for WGPH45A, as this pathway tends to transport hydrophilic, neutral or negatively charged small peptides, and the WGPH45A is further negatively charged (38.27 ± 0.06 in Glu + Gln) [47]. However, it is difficult to consider that the same observed effect in vitro will appear in humans. Hence, once anti-oxidant, anti-inflammatory, and atheroprotective properties have been established, it is necessary to focus on identified bioactive peptides from WGPH45A and to consider performing the appropriate in vivo assays in animal models and clinical trials in order to evaluate their real effect as a nutraceutical.

## 4. Conclusions

**Figure 8:**
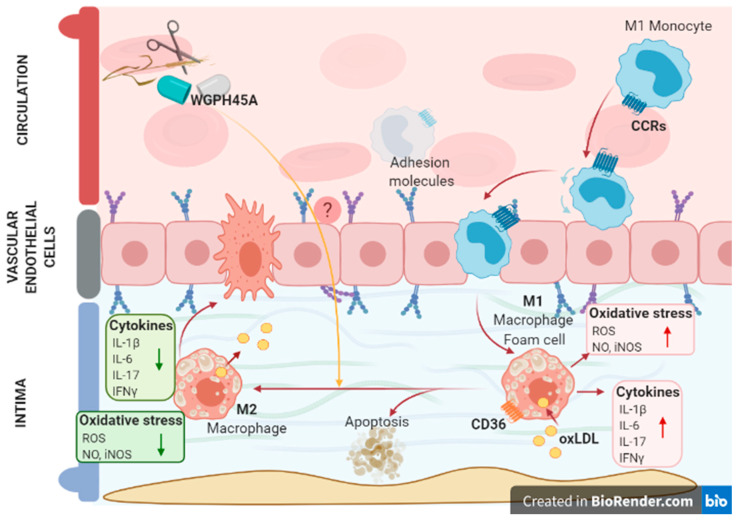
Summary of anti-oxidant, anti-inflammatory, and atheroprotective effects of WGPH45A in primary human monocytes. LPS-stimulated monocytes highly express the M1 phenotype, they used CCRs chemokines to infiltrate the intima, which is assisted by adhesion molecules. Once in the intima, monocytes differentiate into pro-inflammatory macrophages. In the intima, macrophages react to atherogenic lipoproteins (ox-LDL) via pinocytosis by scavenger receptor pathways (CD36), resulting in the foam cell formation. These cells cause oxidative stress and enhance pro-inflammatory cytokine production, which may lead to death by apoptosis in the necrotic core of the plaque. When WGPH45A is added, the foam cell may cause the efflux of ox-LDL and induce the transition to the M2 phenotype, which is characterized by the resolution of inflammation and tissue repair.

In conclusion, WGPH45A showed generally anti-oxidant and anti-inflammatory effects in primary human monocytes as is summarized in Figure 8. Our results imply not only the ROS attenuation, but also the regulation of inflammatory genes leading to an improvement of inflammatory status, which could be used to attenuate or prevent the inflammation in general chronical pathologies. More than that, as enzymes have been shattering the corresponding gluten-epitopes that cause celiac disease, the WGPH could be employed in specific celiac-foods due to specific anti-inflammatory molecule presence in order to mitigate intestinal inflammation. According to in vitro gastrointestinal studies of celiac disease, the immune cell activation depends on the epitopes created during hydrolysis [10]. Because of this, different epitopes may be formed over the reaction progression. However, these epitopes are oligopeptides and are essentially toxic for the cells and induce damage via oxidative stress or the manner of ROS production [10]. Our results have shown a down regulation of oxidative stress genes in human primary monocytes; thereupon the WGPH could not contain these toxic-oligopeptides.

The main topic is the integration between all the outcomes aimed at identifying the nutraceutical value for the hydrolysate of interest. WGHP could be used as an adjuvant therapy to reduce local chronic inflammatory response, which appears in the course of atherosclerosis disease. The WGPH likewise could be recommended for atherosclerosis prevention in patients who are genetically predisposed to it. Because of this, our WGPH demonstrated the ability to down-regulate atherosclerosis-related genes as CCR family genes. This knowledge can develop novel, good-quality, safe WGPH-containing food products or nutraceuticals for sports nutrition, clinical nutrition, and infant formulas. 

## Figures and Tables

**Table 1:** Chemical composition of WGPH45A. Values, showed as a percentage in dry basis, are mean ± standard deviation of triplicates.

(%)	WGPH45A
Protein	81.36 ± 0.44
Ash	6.19 ± 0.15
Fibre	1.51 ± 0.05
Oil	0.92 ± 0.04
Soluble sugars	0.01 ± 0.00
Polyphenols	0.06 ± 0.01
Other compounds	9.95 ± 0.63

**Table 2:** Amino acid composition of WGPH45A. Values, showed as a percentage of total amino acid content, are mean ± standard deviation of triplicates.

Amino Acid	WGPH45A	FAO/WHO
Aspartic acid + asparragine	4.03 ± 0.09	
Glutamic acid + Glutamine	38.27 ± 0.06	
Serine	5.73 ± 0.03	
Histidine	1.98 ± 0.01	1.9
Glycine	3.70 ± 0.11	
Threonine	3.05 ± 0.02	3.4
Arginine	3.69 ± 0.03	
Alanine	3.03 ± 0.02	
Proline	7.12 ± 0.63	
Tyrosine	3.49 ± 0.14	
Valine	4.03 ± 0.04	3.5
Methionine	0.94 ± 0.14	2.5 ^a^
Cysteine	1.60 ± 0.24	
Isoleucine	3.67 ± 0.00	2.8
Tryptophan	0.87 ± 0.00	
Leucine	7.40 ± 0.01	6.6
Phenylalanine	5.20 ± 0.02	6.3 ^b^
Lysine	2.20 ± 0.15	5.8

^a^ Metionine + Cysteine; ^b^ Phenilalanine + Tyrosine.

## Supplementary Materials

The following are available online at https://www.mdpi.com/2304-8158/9/7/854/s1, Table S1: Chemical composition of WGPC. Values, presented as percentage in dry basis, are mean ± standard deviation of triplicates, Table S2: Amino acid composition of WGPC. Values, presented as percentage of amino acids on total amino acid content, are mean ± standard deviation of triplicates, Table S3: Sequence and GenBank accession number of oligonucleotides used in RT-qPCR.
