# Supplementary material for: Evaluation of Anti-Inflammatory and Atheroprotective Properties of Wheat Gluten Protein Hydrolysates in Primary Human Monocytes
Source: Foods. 2020 Jun 30;9(7):854. doi: 10.3390/foods9070854 (PMC7404777; doi:10.3390/foods9070854)
Supplement: Supplementary file 1 [file foods-09-00854-s001.pdf]

Suppementary Materials:

Table S1. Chemical composition of WGPC. Values, presented as percentage in dry basis, are mean ± standard deviation of triplicates.

(%)	WGPC
Protein	89.24 ± 0.06
Ash	0.92 ± 0.20
Fibre	2.43 ± 0.22
Oil	1.47 ± 0.08
Soluble sugars	0.55 ± 0.01
Polyphenols	0.02 ± 0.00
Others[a]	5.37

[a] measured as 100 - (protein-ash-fibre-oil-soluble sugars-polyphenols).

Table S2. Amino acid composition of WGPC. Values, presented as percentage of amino acids on total amino acid content, are mean ± standard deviation of triplicates.

Amino Acid	WGPC
Aspartic acid + asparragine	4.54 ± 0.02
Glutamic acid + Glutamine	40.71 ± 0.13
Serine	6.46 ± 0.10
Histidine	2.10 ± 0.03
Glycine	4.63 ± 0.08
Threonine	2.52 ± 0.01
Arginine	3.61 ± 0.03
Alanine	3.21 ± 0.12
Proline	6.70 ± 0.05
Tyrosine	3.94 ± 0.03
Valine	2.11 ± 0.17
Methionine	1.39 ± 0.16
Cysteine	2.31 ± 0.05
Isoleucine	1.87 ± 0.04
Tryptophan	0.49 ± 0.03
Leucine	6.51 ± 0.00
Phenylalanine	5.40 ± 0.20
Lysine	1.80 ± 0.07

Table S3. Sequence and GenBank accession number of oligonucleotides used in RT-qPCR.

Target	GenBank Accession Number	Direction	Sequence (5′→3′)
CCR2	NM_001123041.2	Forward	GTGTGTGGAGGTCCAGGAGT
		Reverse	AAGCCAGACGTGTGATTTCC
CCR5	NM_009917	Forward	CAGATGGCTTCTCCACACAA
		Reverse	CGGAGCTTGAGAAAAACCAG
CCR7	NM_001301716.1	Forward	TGGAGGCCTTTATCACCATC
		Reverse	TGTAGGGCAGCTGGAAGACT
CD36	NM_ 001159556.1	Forward	CAGCAAGGCCAGATATCACA
		Reverse	GAGCTATGCAGCATGGAACA
HO-1	NM_010442	Forward	CACGCATATACCCGCTACCT
		Reverse	CCAGAGTGTTCATTCGAGCA
IFNγ	NM_000619.2	Forward	TCCCATGGGTTGTGTGTTTA
		Reverse	AAGCACCAGGCATGAAATCT
IL-1β	NM_000576.2	Forward	CTGTCCTGCGTGTTGAAAGA
		Reverse	TTCTGCTTGAGAGGTGCTGA
IL-6	NM_000600.4	Forward	TACCCCCAGGAGAAGATTCC
		Reverse	TTTTCTGCCAGTGCCTCTTT
IL-17	NM_002190.2	Forward	ACCAATCCCAAAAGGTCCTC
		Reverse	ACCAATCCCAAAAGGTCCTC
iNOS	NM_000625.4	Forward	ACAAGCCTACCCCTCCAGAT
		Reverse	TCCCGTCAGTTGGTAGGTTC
GAPDH	NM_002046.6	Forward	GAGTCAACGGATTTGGTCGT
		Reverse	GACAAGCTTCCCGTTCTCAG
HPRT	NM_000194	Forward	ACCCCACGAAGTGTTGGATA
		Reverse	AAGCAGATGGCCACAGAACT
